# Bilateral thoracic disc herniation with abdominal wall paresis: a case report

**DOI:** 10.1007/s00701-020-04431-5

**Published:** 2020-06-04

**Authors:** Vicki Marie Butenschoen, Lisa Hoenikl, Marcus Deschauer, Bernhard Meyer, Jens Gempt

**Affiliations:** 1 School of Medicine, Klinikum rechts der Isar, Neurosurgical Department, Technical University of Munich, Munich, Germany; 2 School of Medicine, Klinikum rechts der Isar, Neurological Department, Technical University of Munich, Munich, Germany

**Keywords:** Disc herniation, Motor deficit, Thoracic spine

## Abstract

We present a rare case of a patient initially presenting with unilateral abdominal wall bulging and radicular pain caused by a lateral disc herniation at Th11/12, later suffering from a hernia recurrence with bilateral disc prolapse and motor deficits. The patient underwent sequesterectomy via a right hemilaminectomy at Th11, and after 8 weeks, a bilateral sequesterectomy with semirigid fusion Th11/12 was performed. Unilateral motor deficits at the thoracic level have been discussed in case reports; a bilateral disc protrusion with abdominal wall bulging occurring as a recurrent disc herniation has never been described before.

## Background

While lumbar disc herniation present a very common pathology in patients between 30 and 50 years of age with an incidence of 5 to 20 cases per 1000 adults annually [6], thoracic disc herniation occurs more rarely [2, 16] and accounts for less than 4% of the surgeries performed [2].

Compared to cervical and lumbar disc protrusions, thoracic disc herniation seldom causes symptoms. The fraction of symptomatic disc prolapses in the thoracic spine amounts to only 0.1 to 3% of all spinal disc herniation [2].

Symptomatic thoracic disc herniation mainly causes signs of myelopathy (50 to 80%) [7, 8, 14], thoracic or abdominal radiculopathy and abdominal pain [1, 15], or, in rare cases, anterior spinal artery syndrome [17]. Motor deficits at the thoracic level with abdominal wall bulging due to a paresis of the musculus obliquus abdominis are currently only a matter of case reports, up now to only four cases, with unilateral disc prolapses that have been described in the literature [9, 12, 20, 25]. We hereby present a rare case of a patient initially presenting with unilateral abdominal pain and abdominal wall hernia, with complete neurophysiological assessment, intraoperative imaging, and postoperative outcome, who later developed a bilateral recurrent disc protrusion with bilateral radiculopathy and symmetrical abdominal wall bulging due to muscle paresis.

## Case presentation

### Case history

**Fig. 1:**
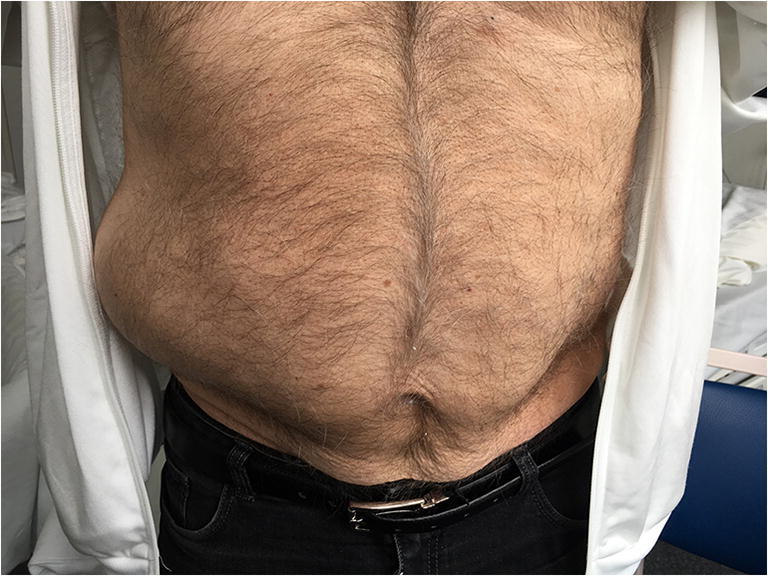
Initial presentation of the patient with abdominal wall bulging right-sided

An 81-year-old patient presented with sudden onset bulging of his right abdominal wall (Fig. 1) and radicular pain projected to the Th11 dermatome. The colleagues of general and visceral surgery rejected the initially suspected diagnosis of an abdominal hernia. Sensibility was intact, and the patient did not suffer from any pain radiating into his legs. Past medical history included a stroke of the left middle cerebral artery (MCA) in 2015 and high blood pressure.

### Physical examination results

Except for the right abdominal wall bulging and radicular pain projecting on the Th11 nerve root, no focal neurological deficit could be detected. There were no clinical signs of myelopathy, no burdening back pain, no bladder or sphincter dysfunction, and no gait impairment. Reflexes were minimally prominent on the right side due to the history of a left stroke.

### Imaging

**Fig. 2:**
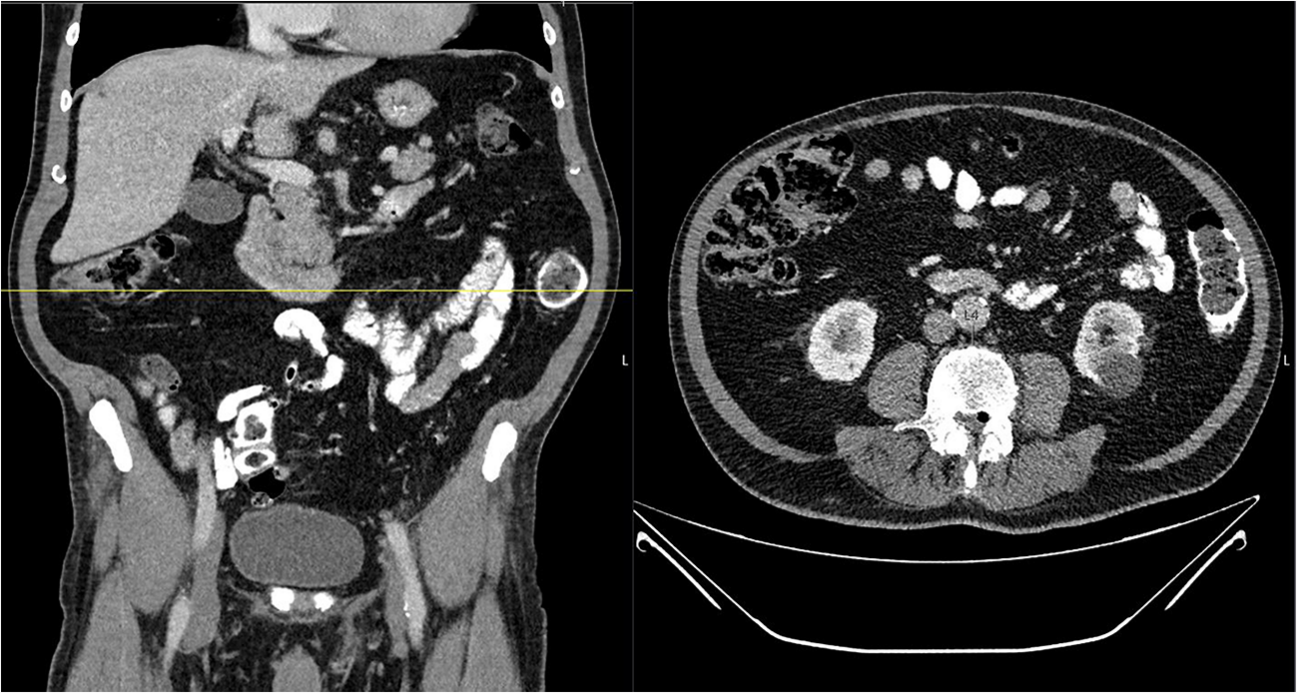
The abdominal CT scan excludes the suspected diagnosis of an abdominal wall herniation

**Fig. 3:**
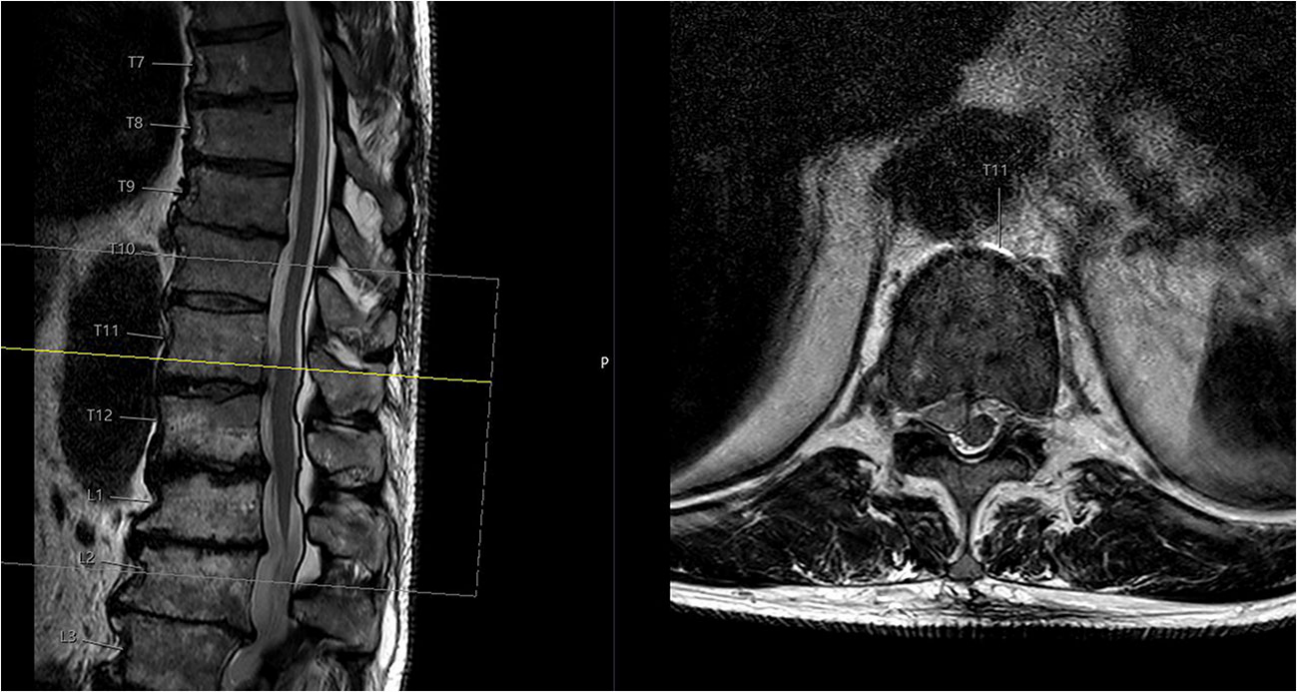
The MRI scan of the thoracolumbar spine T2 weighted axial and sagittal presents a right-sided disc herniation TH 11/12

At the initial presentation at the general and visceral surgical department, the patient underwent computer tomography (CT) of the abdomen. An abdominal wall hernia was excluded (Fig. 2). After neurological examination, an MRI of the thoracic and lumbar spine was performed, showing a large disc prolapse at the Th11/12 level on the right side affecting the right Th11 nerve root (Fig. 3).

### Electrophysiology

Needle electromyography (EMG) of the right-sided Th11 paravertebral muscles 8 days after onset revealed fibrillations as a sign of florid denervation, indicating acute Th11 nerve root compression.

### Surgical treatment

Due to the acute denervation and persistent pain symptoms, the disc herniation was removed 6 days after symptom onset through a Th11 hemilaminectomy on the right side with intraoperative X-ray control.

### Outcome and second operation

The patient was discharged on the second postoperative day with moderate wound pain and complete resolving of the abdominal radicular pain. The abdominal wall bulging did not resolve.

**Fig. 4:**
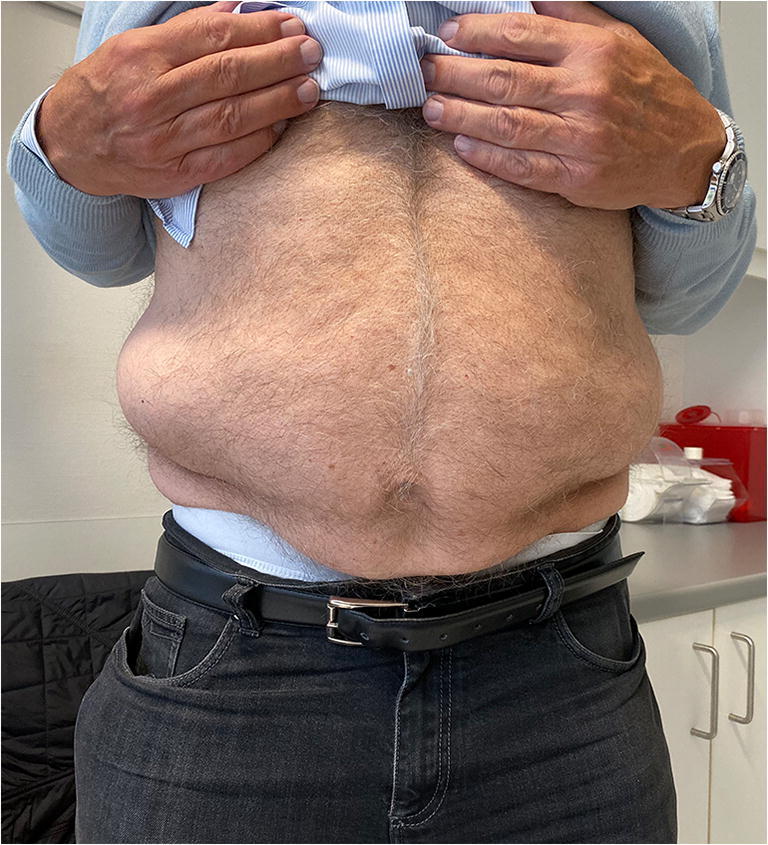
In the clinical follow-up 8 weeks after the first operation, the patient presented with bilateral abdominal wall bulging

**Fig. 5:**
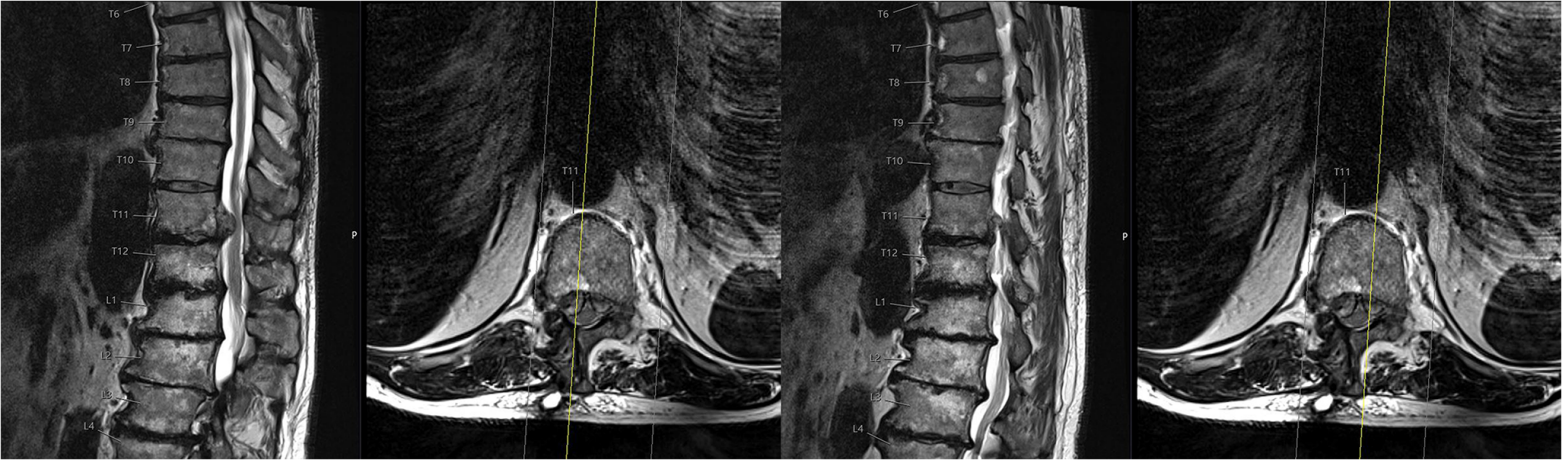
MRI of the thoracolumbar spine T2 weighted 8 weeks after initial presentation with clinical bilateral thoracic abdominal wall bulging and a bilateral relapse of disc herniation Th11/12

After 8 weeks, the patient presented in the outpatient department with a new sudden onset of abdominal bulging occurring on the left side (symmetrical to the already existing right wall bulging: Fig. 4). The MRI revealed a recurrent disc prolapse at the operated Th11/12 level—now on both sides and accentuated on the right side—as well as a degeneration of the Th11/12 Modic type I disc, matching the clinical symptoms of the patient (Fig. 5). EMG of the left-sided Th11 paravertebral muscles 4 days after the onset of left abdominal wall paresis was normal, probably because of too early an examination.

**Fig. 6:**
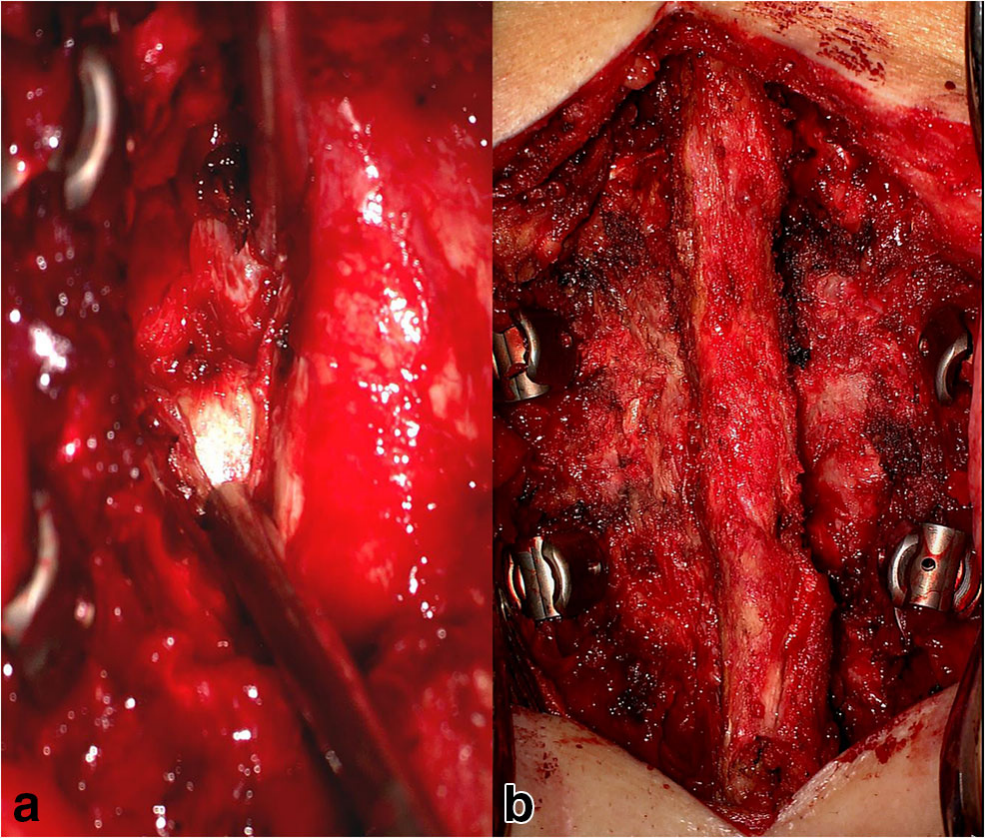
Intraoperative findings: A discectomy (**a**) and dynamic fusion (**b**) was performed

**Fig. 7:**
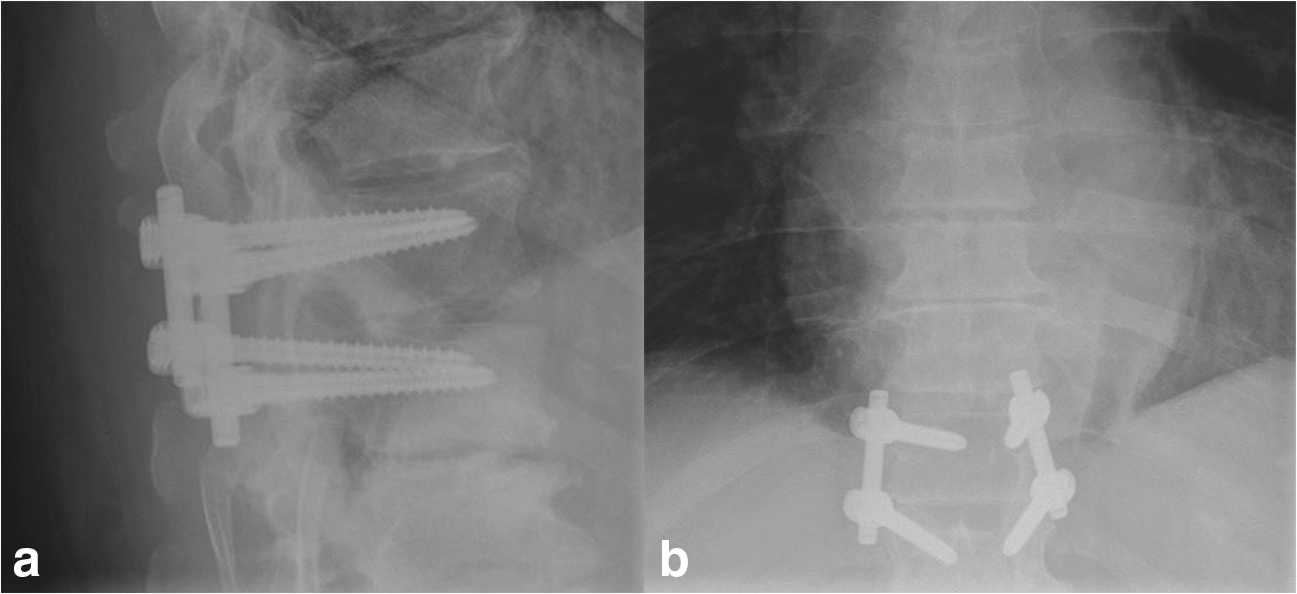
Postoperative X-ray in lateral (**a**) and anterior-posterior imaging (**b**)

Due to the already performed hemilaminectomy on the right side and the symptoms of segmental instability, the patient was advised to have an operative treatment with discectomy and Th11/12 dynamic fusion with a semirigid (Cosmic MIA, Ulrich) instrumentation (Figs. 6 and 7). Back pain resolved after the operative treatment; the bilateral abdominal wall bulging remained stable (follow-up 5 months).

## Discussion and conclusions

### Diagnostic pathway

Important differential diagnoses include thoracic diabetic radiculopathy [10], herpes zoster infection [18], abdominal malignancies, prior laparoscopic or minimally invasive surgeries [3, 22], and abdominal wall hernia and can lead to a swelling and bulging of the abdominal wall with radiating thoracic pain. These entities were considered and excluded via a paraspinal EMG and a CT scan. An MRI scan was performed leading to the diagnosis of thoracic disc herniation. In fact, patients may confound the abdominal pain with visceral pathologies, which is more common than disc prolapses causing the symptoms. The diagnostic pathway is indeed explainable, as symptomatic thoracic disc herniation is rare, accounting for only 0.25–0.57% of all disc herniation [24], and is more commonly above the level TH8 [24]. A further supplementary diagnostic method performed in this case was the EMG, showing signs of mono-segmental nerve root compression [11].

### Initial surgical procedure

There are several possible surgical approaches to treat thoracic disc herniation, depending on localization, calcification of the herniated disc, and segmental instability. Posterolateral approaches are recommended for soft lateral disc herniation [8], and the transthoracic approach is more commonly used in large calcified central hernia [11]. Whether to stabilize or to perform a discectomy at all is currently a matter of debate [13, 21, 23]. Instrumented fusion can be required in cases performed from the posterior [11]. As our patient initially presented with an acute unilateral soft disc herniation without signs of segmental instability, we opted for a posterior approach with instrumentation.

### Recurrent disc herniation

Less data is available on the recurrence rate of thoracic disc herniation. The general rate of recurrent disc herniation has been reported between 0.5–25%, especially in the first months after a successful first surgical procedure [5], but describes only recurrent disc herniation in the lumbar spine. Some of the common possible risk factors are obesity, smoking, male gender, diabetes, weightlifting, the size of the annular tear, and type of primary operation [5].

### Surgical procedure after recurrent disc herniation

Currently, we lack guidelines with significant comparative studies for the surgical treatment of recurrent disc herniation. In a systematic review comparing possible treatments after recurrent lumbar disc herniation [5], excellent outcomes were described for re-discectomies, posterolateral fusion, and posterior lumbar interbody fusion (PLIF) without significant differences between the applied options.

Dower et al. found similar rates of satisfactory outcomes in patients undergoing discectomy alone versus discectomy with fusion (79.9% vs. 77.8%, respectively) but stated significant improvements in back pain scores in patients undergoing fusion compared with isolated discectomy (60.1% vs. 47.2%, respectively) [4]. Currently, many surgeons advocate for re-discectomy alone in cases without deformity, instability, or associated back pain and opt for instrumented fusion if one of the symptoms is present [19].

### Patient outcome

The clinical outcome after disc or recurrent disc herniation is dependent on several factors, such as the time between first symptoms to surgical approach, size, and location of disc herniation, and preoperative clinical status. Most studies focus on the clinical outcome in myelopathic patients [8], and we currently lack sufficient data describing regeneration of thoracic motor deficits. In our case, the abdominal wall bulging did not resolve after surgical treatment.

## Conclusion

The patient showed a clear benefit from the two operations regarding the radicular and back pain, which were distinctly better than the pain was preoperatively. The motor deficit with the bilateral abdominal wall bulging remained stable. To our knowledge, unilateral abdominal wall paresis due to thoracic disc herniation has only been reported in four case reports; a bilateral disc protrusion with symmetrical abdominal wall paresis has never been described before. We hereby present a unique and very rare case of motor deficits at the thoracic level without myelopathy, with diagnostic implications, surgical treatment, and clinical outcome.

## References

[1] Baranto A, Börjesson M, Danielsson B, Hellström M, Swärd L (2009). Acute chest pain in a top soccer player due to thoracic disc herniation. Spine (Phila Pa 1976).

[2] Court C, Mansour E, Bouthors C (2018). Thoracic disc herniation: surgical treatment. Orthop Traumatol Surg Res.

[3] Dakwar E, Le TV, Baaj AA, Le AX, Smith WD, Akbarnia BA, Uribe JS (2011). Abdominal wall paresis as a complication of minimally invasive lateral transpsoas interbody fusion. Neurosurg Focus.

[4] Dower A, Chatterji R, Swart A, Winder MJ (2016). Surgical management of recurrent lumbar disc herniation and the role of fusion. J Clin Neurosci.

[5] Drazin D, Ugiliweneza B, Al-Khouja L, Yang D, Johnson P, Kim T, Boakye M (2016). Treatment of recurrent disc herniation: a systematic review. Cureus.

[6] Fjeld OR, Grøvle L, Helgeland J, Småstuen MC, Solberg TK, Zwart JA, Grotle M (2019). Complications, reoperations, readmissions, and length of hospital stay in 34 639 surgical cases of lumbar disc herniation. Bone Joint J.

[7] Hott JS, Feiz-Erfan I, Kenny K, Dickman CA (2005). Surgical management of giant herniated thoracic discs: analysis of 20 cases. J Neurosurg Spine.

[8] Kang J, Chang Z, Huang W, Yu X (2018). The posterior approach operation to treat thoracolumbar disc herniation: a minimal 2-year follow-up study. Medicine.

[9] LaBan MM, Gorin G (2007). A thoracic disc herniation presenting as an abdominal hernia. Am J Phys Med Rehabil.

[10] Lempert T, Skotzek B (1988). Abdominal wall paresis in thoracic diabetic neuropathy. Nervenarzt.

[11] Li W, Liu Y-C, Zheng C-F, Miao J, Chen H, Quan H-Y, Yan S-H, Zhang K (2018). Diagnosis of compressed nerve root in lumbar disc herniation patients by surface electromyography. Orthop Surg.

[12] Meyer F, Feldmann H, Töppich H, Celiker A (1991). Unilateral paralysis of the abdominal wall musculature caused by thoracic intervertebral disk displacement. Zentralbl Neurochir.

[13] Oitment C, Kwok D, Steyn C (2019). Calcified thoracic disc herniations in the elderly: revisiting the laminectomy for single level disease. Global Spine J.

[14] Oppenlander ME, Clark JC, Kalyvas J, Dickman CA (2013). Surgical management and clinical outcomes of multiple-level symptomatic herniated thoracic discs. J Neurosurg Spine.

[15] Ozturk C, Tezer M, Sirvanci M, Sarier M, Aydogan M, Hamzaoglu A (2006). Far lateral thoracic disc herniation presenting with flank pain. Spine J.

[16] Quint U, Bordon G, Preissl I, Sanner C, Rosenthal D (2012). Thoracoscopic treatment for single level symptomatic thoracic disc herniation: a prospective followed cohort study in a group of 167 consecutive cases. Eur Spine J.

[17] Reynolds JM, Belvadi YS, Kane AG, Poulopoulos M (2014). Thoracic disc herniation leads to anterior spinal artery syndrome demonstrated by diffusion-weighted magnetic resonance imaging (DWI): a case report and literature review. Spine J.

[18] Santiago-Perez S, Nevado-Estevez R, Perez-Conde MC (2012). Herpes zoster-induced abdominal wall paresis: neurophysiological examination in this unusual complication. J Neurol Sci.

[19] Shepard N, Cho W (2019). Recurrent lumbar disc herniation: a review. Global spine journal.

[20] Stetkarova I, Chrobok J, Ehler E, Kofler M (2007). Segmental abdominal wall paresis caused by lateral low thoracic disc herniation. Spine (Phila Pa 1976).

[21] Telfeian AE, Jasper GP, Oyelese AA, Gokaslan ZL (2016). Technical considerations in transforaminal endoscopic spine surgery at the thoracolumbar junction: report of 3 cases. Neurosurg Focus.

[22] van Ramshorst GH, Kleinrensink GJ, Hermans JJ, Terkivatan T, Lange JF (2009). Abdominal wall paresis as a complication of laparoscopic surgery. Hernia.

[23] Wagner R, Telfeian AE, Iprenburg M, Krzok G, Gokaslan Z, Choi DB, Pucci FG, Oyelese A (2016). Transforaminal endoscopic foraminoplasty and discectomy for the treatment of a thoracic disc herniation. World Neurosurg.

[24] Yi S, Kim SH, Shin HC, Kim KN, Yoon DH (2007). Outcome of surgery for a symptomatic herniated thoracic disc in relation to preoperative characteristics of the disc. Acta Neurochir.

[25] Zambelis T, Polydorou A, Anagnostou E, Angourakis P, Vassilopoulou S (2018) Unusual presentation of thoracic disc herniation. Br J Neurosurg:1–2. 10.1080/02688697.2018.1466996 10.1080/02688697.2018.1466996 29688067

