# Correction to: Bilateral thoracic disc herniation with abdominal wall paresis: a case report

**DOI:** 10.1007/s00701-021-04898-w

**Published:** 2021-06-23

**Authors:** Vicki Marie Butenschoen, Lisa Hoenikl, Marcus Deschauer, Bernhard Meyer, Jens Gempt

**Affiliations:** 1 School of Medicine, Klinikum rechts der Isar, Neurosurgical Department, Technical University of Munich, Munich, Germany; 2 School of Medicine, Klinikum rechts der Isar, Neurological Department, Technical University of Munich, Munich, Germany

**Correction to: Acta Neurochirurgica (2020) 162:2055–2059**

10.1007/s00701-020-04431-5

The article “Bilateral thoracic disc herniation with abdominal wall paresis:a case report”, written by Butenschoen, V.M., Hoenikl, L., Deschauer, M,. Meyer, B., & Gempt, J., was originally published Online First without Open Access. After publication in volume 162, issue 9, page 2055–2059 the author decided to opt for Open Choice and to make the article an Open Access publication. Therefore, the copyright of the article has been changed to © The Author(s) 2020 and the article is forthwith distributed under the terms of the Creative Commons Attribution 4.0 International License, which permits use, sharing, adaptation, distribution and reproduction in any medium or format, as long as you give appropriate credit to the original author(s) and the source, provide a link to the Creative Commons licence, and indicate if changes were made. The images or other third party material in this article are included in the article’s Creative Commons licence, unless indicated otherwise in a credit line to the material. If material is not included in the article’s Creative Commons licence and your intended use is not permitted by statutory regulation or exceeds the permitted use, you will need to obtain permission directly from the copyright holder. To view a copy of this licence, visit http://creativecommons.org/licenses/by/4.0. Open access funding enabled and organized by Projekt DEAL.

